# Simultaneous Determination of Benzene and Toluene in Pesticide Emulsifiable Concentrate by Headspace GC-MS

**DOI:** 10.1155/2013/121783

**Published:** 2013-03-31

**Authors:** Lidong Cao, Hua Jiang, Jing Yang, Li Fan, Fengmin Li, Qiliang Huang

**Affiliations:** ^1^Institute of Plant Protectsion, Chinese Academy of Agricultural Sciences, Key Laboratory of Integrated Pest Management in Crops of Ministry of Agriculture, Beijing 100193, China; ^2^Institute of Quality Standard and Testing Technology for Agro-Products, Chinese Academy of Agricultural Sciences, Beijing 100081, China

## Abstract

The toxic inert ingredients in pesticide formulations are strictly regulated in many countries. In this paper, a simple and efficient headspace-gas chromatography-mass spectrometry (HSGC-MS) method using fluorobenzene as an internal standard (IS) for rapid simultaneous determination of benzene and toluene in pesticide emulsifiable concentrate (EC) was established. The headspace and GC-MS conditions were investigated and developed. A nonpolar fused silica Rtx-5 capillary column (30 m × 0.20 mm i.d. and 0.25 **μ**m film thickness) with temperature programming was used. Under optimized headspace conditions, equilibration temperature of 120°C, equilibration time of 5 min, and sample size of 50 **μ**L, the regression of the peak area ratios of benzene and toluene to IS on the concentrations of analytes fitted a linear relationship well at the concentration levels ranging from 3.2 g/L to 16.0 g/L. Standard additions of benzene and toluene to blank different matrix solutions 1ead to recoveries of 100.1%–109.5% with a relative standard deviation (RSD) of 0.3%–8.1%. The method presented here stands out as simple and easily applicable, which provides a way for the determination of toxic volatile adjuvant in liquid pesticide formulations.

## 1. Introduction

Emulsifiable concentrate (EC) formulations, which are the blend of active ingredient, organic solvent, and surfactants, are the major pesticide formulations currently used in many developing countries. Compared to other formulations, advantages of EC include higher concentration of active ingredient and being simple to manufacture and low in cost, relatively easy to handle, transport, and store, not abrasive, and uniformly spreading and wetting under normal spray and weather conditions. However, the disadvantages of EC cannot be overlooked. The large amount of the organic solvent makes the EC formulations flammable and corrosive. More seriously, the hidden toxic inert ingredients in EC, such as benzene and toluene, might cause adverse effects to humans and the environment. 

Benzene and toluene are ubiquitous environmental pollutants. The American Petroleum Institute (API) stated in 1948 that “it is generally considered that the only absolutely safe concentration for benzene is zero” [1]. The International Agency for Research on Cancer (IARC) rated benzene as “known to be carcinogenic to humans” (group 1) [2]. Although toluene is less toxic than benzene, inhaling high levels of toluene in a short time may cause lightheadedness, nausea, or sleepiness. It can also cause unconsciousness and even death. In order to reduce the potential risk posed to human health and the environment, the Unites States Environmental Protection Agency (USEPA) has divided the approximately 1,200 intentionally added inert ingredients currently contained in pesticide products into four toxicity categories (Lists 1–4). According to the classification, benzene belongs to List 1 (inerts of toxicological concern), and toluene belongs to List 2 (potentially toxic inerts) [3]. The Canada Health Pest Management Regulatory Agency (PMRA) has also implemented a similar program on the regulations of the inert ingredient in pesticide products [4]. In China, the research and regulation on the safety of pesticide adjuvants have just started, and the corresponding policymaking is on the agenda. Therefore, it is highly desirable to establish sensitive and accurate analytical methods for the determination of the toxic adjuvants, especially for the high-priority pollutant benzene.

Regarding the complex matrix of samples, for the volatile toxic organic chemicals, headspace-gas chromatography (HSGC) analysis which allows direct injection of specimens, is without doubt the most common procedure used [5]. In recent years, coupled with different detectors, utilizing novel techniques of sample introduction and pretreatment, like solid-phase microextraction (SPME), HSGC analysis has been developed for the determination of benzene and toluene in different sample matrices, for example, water [6], soil [7, 8], human tissue [9], soft drinks [10], pharmaceutical products [11], and so forth. However, there are little reports about the HSGC analysis of benzene and toluene in pesticide formulations [12]. Recently, we established a rapid HSGC method using flame ionization detector (FID) for the determination of methanolin pesticide EC [13]. Almost at the same time, Liu reported similar HSGC-FID method for the determination of benzene and seven harmful pesticide adjuvants, mainly polyhalohydrocarbon in pesticide EC [12]. Both our and Liu's results indicated that the obvious matrix effects cannot be avoided by hand injection of the vapor in sealed headspace bottle using an airtight syringe with or without internal standard. To address this problem, in this paper we report a rapid HSGC-mass spectrometry (HSGC-MS) method for simultaneous assay of benzene and toluene in pesticide EC. The matrix effects can be basically eliminated by tuning the equilibration temperature and sample size with a headspace autosampler system.

The advantages of HSGC-MS are threefold [9]. As a homogeneous gas sample is introduced into the gas chromatograph, the method permits the determination of volatile present in an essentially nonvolatile matrix which may be difficult to analyse directly or would otherwise require sample extraction or preparation. The second advantage consists in the proper selection of equilibrium conditions, mainly the temperature, so that the volatile concentration can be measured in the headspace, rendering easier the determination of trace concentrations in the sample. The third benefit rests with using the mass spectrometry in selected ion monitoring (SIM) model which increase, the sensitivity and specificity (masses of no interest are ignored).

Taking chlorpyrifos EC as a model and fluorobenzene as an internal standard, the objective of the present work was to develop a simple and efficient HSGC-MS method for rapid simultaneous determination of benzene and toluene in pesticide EC. The headspace parameters (equilibration temperature, and equilibration time) and method validation were also explored.

## 2. Experimental

### 2.1. Chemicals

Benzene, toluene, and xylene, all of analytical grade with purity ≥99.0%, were purchased from Beijing Chemical Works (Beijing, China). Analytical purity fluorobenzene was supplied by Sinopharm Chemical Reagent Co., Ltd. (Shanghai, China). Chlorpyrifos technical with purity 98.0% was provided by Dow AgroSciences (USA). Tween-80 (polysorbate) was purchased from Xilong Chemical Co., Ltd. (Guangzhou, China). Beta-Cypermethrin technical with purity 95.0% was provided by Nanjing Ronch Chemical Co., Ltd.

### 2.2. Instrumentation

Automated analysis was performed using a CTC Combi Pal Headspace AOC-5000 autosampler (Zwingen, Switzerland) controlled by a Cycle Composer software. GC-MS analysis was performed by a GCMS-QP2010 gas chromatography mass spectrometer (Shimadzu, Kyoto, Japan), equipped with a split/splitless injector and nonpolar fused silica Rtx-5 capillary column (30 m × 0.20 mm i.d.) with a 0.25-*μ*m film thickness (Restek, Bellefonte, PA). Data were acquired through GC-MS solution software (Shimadzu, Japan). 

### 2.3. HSGC-MS Analysis

Aliquots of 50 *μ*L sample solutions were added to 20 mL screw cap headspace vials. The vials then were placed in the headspace sample tray under the following operating conditions: equilibration time of 5 min with agitation, incubation temperature of 120°C; 100 *μ*L of the vapor in vials was transferred to GC injection port with 1.0 mL syringe heated at 140°C by AOC-5000 robotic arm for GC-MS analysis.

The injection temperature was 200°C and operated in split mode (1 : 100). Helium was used as the carrier gas with a flow rate of 0.9 mL/min at a constant linear velocity (34.3 cm/s). Initial column temperature was 40°C held for 5 min, programmed at 45°C/min to 230°C, and finally held at 230°C for 5 min. Total run time was 14.7 min.

For the mass spectrometry detection experiments, electron ionization was used with temperatures of 200°C and 250°C for ion source and interface, respectively. The scan range of mass-to-charge ratio (m/z) of ion was 45–110, and quantification analysis was acquired in selected ion monitoring (SIM) mode. Fragment ions were monitored for each analyte as specified in the following: m/z 78, 77, and 52 (benzene); 96, 70, and 50 (fluorobenzene); 91, 92, and 65 (toluene). 

### 2.4. Sample Preparation

Stock solution A: accurately weighed 200.0 g of chlorpyrifos technical, 50.0 g of Tween-80, and 250.0 g of xylene to 1000 mL of erlenmeyer flask were dissolved under ultrasound to afford 40.0% (w/w) chlorpyrifos EC. 

Stock solution B: accurately weighed 80.0 g of chlorpyrifos technical and 120.0 g of xylene to 500 mL of erlenmeyer flask were dissolved under ultrasound to afford 40.0% (w/w) chlorpyrifos solution in xylene. 

Stock solution C: accurately weighed 20.0 g of Tween-80, and 180.0 g of xylene to 500 mL of erlenmeyer flask were dissolved under ultrasound to afford 10.0% (w/w) Tween-80 solution in xylene. 

Stock solution D: accurately weighed 5.0 g of beta-cypermethrin technical, 20.0 g of Tween-80, and 175.0 g of xylene to 500 mL of erlenmeyer flask were dissolved under ultrasound to afford 2.5% (w/w) beta-cypermethrin EC. 

Stock internal standard (IS) solution: accurately weighed 5.0 g of fluorobenzene into 50.0 mL volumetric flask was made up to constant volume with stock solution A to afford 0.1 g/mL stock IS solution.

## 3. Results and Discussion

### 3.1. Internal Standard

**Figure 1:**
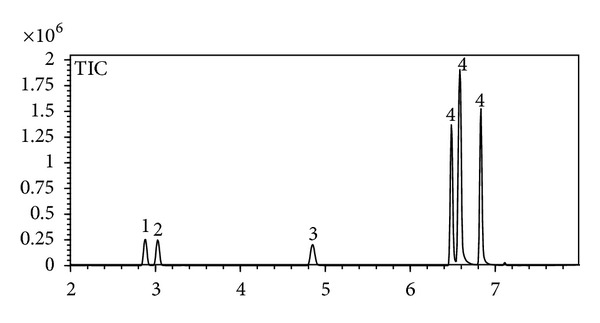
Total ion chromatogram of chlorpyrifos EC spiked with 4.7 g/L of benzene, 4.3 g/L of fluorobenzene, and 4.7 g/L of toluene (equilibration temperature of 120°C, sample size of 20 *μ*L; 1, benzene; 2, fluorobenzene; 3, toluene; 4, xylene).

The effect of the matrix composition has long been regarded as a crucial problem in quantitative headspace analysis. In order to compensate for this effect, and to obtain accurate results, analysts often use the internal standard technique for calibration [14]. Later, Drozd et al. found that due to the rather complex nature of the headspace equilibria, internal standard calibration does not generally eliminate the matrix effect [15]. However, this technique can dramatically reduce the imprecision in injected sample volume measurements and other experimental variables. Therefore, during our investigation, structurally similar fluorobenzene having comparable physical properties with the analytes benzene and toluene was selected as an internal standard.  Figure 1 clearly shows that IS fluorobenzene separates well with the analytes benzene and toluene. Furthermore, the method demonstrated excellent chromatographic specificity with no endogenous interference at the retention times of benzene, toluene, and IS (2.88, 4.85, and 3.03, min resp.). 

### 3.2. Equilibration Temperature and Time

Equilibration is the most important headspace sampling step. Careful attention toward choosing appropriate equilibration temperatures and times during method development helps ensuring a robust and long-lived procedure. Headspace sampling temperature dependencies lead to the general use of elevated equilibration temperatures. Higher equilibration temperatures are desirable for both greater headspace sensitivity and shorter equilibration times. What is more important is that higher equilibration temperatures can decrease the matrix effect to some extent. Based on this principle, full evaporation HS-GC technique, which adopts an adequate small sample size, was developed to overcome the matrix effect [16]. 

**Figure 2:**
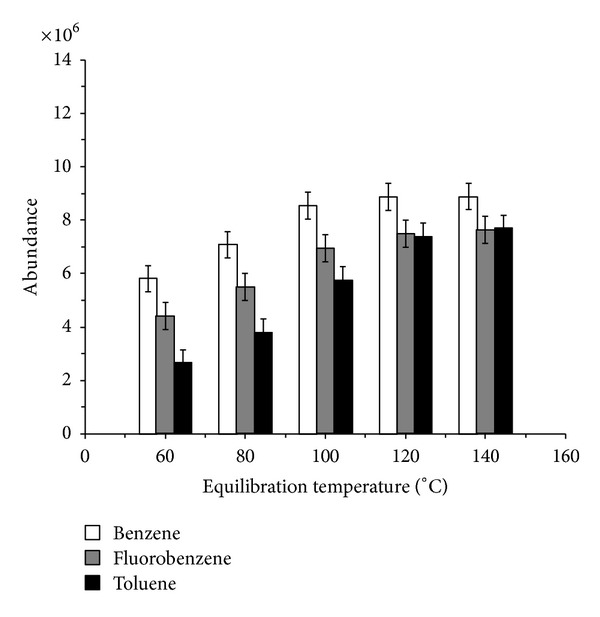
Effect of equilibration temperature on benzene, toluene, and IS peak abundance (equilibration time: 10 min).

**Figure 3:**
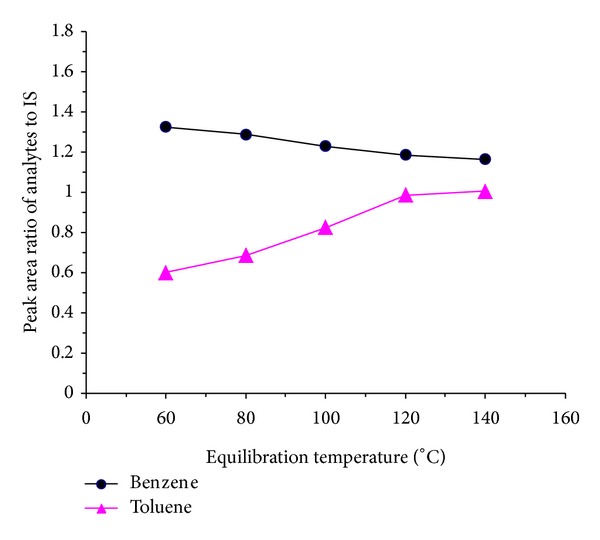
Effect of equilibration temperature on the peak area ratios of benzene and toluene to IS (equilibration time: 10 min).

 Accurately weighed 46.8 mg of benzene, 42.5 mg of toluene, and 47.3 mg of fluorobenzene to 10 mL of volumetric flask were made up to constant volume with stock solution A to afford solution for optimization of headspace conditions. To choose the optimal temperature of analysis, five different equilibration temperatures of 60, 80, 100, 120, and 140°C were tested. The equilibration time was set to 10 min. In Figure 2, the peak abundances are reported versus conditioning temperature. It can be seen that response signals of benzene, toluene, and IS fluorobenzene increase with elevated incubation temperature. However, all the signals reach a plateau after 120°C, which indicates that a near-complete mass transfer of benzene, toluene, and IS to headspace happened, and the matrix effect can be basically eliminated at this temperature. As shown in Figure 3, the peak area ratios Area_(benzene)_/Area_(IS)_ and Area_(toluene)_/Area_(IS)_ also reach constant value after 120°C.

**Figure 4:**
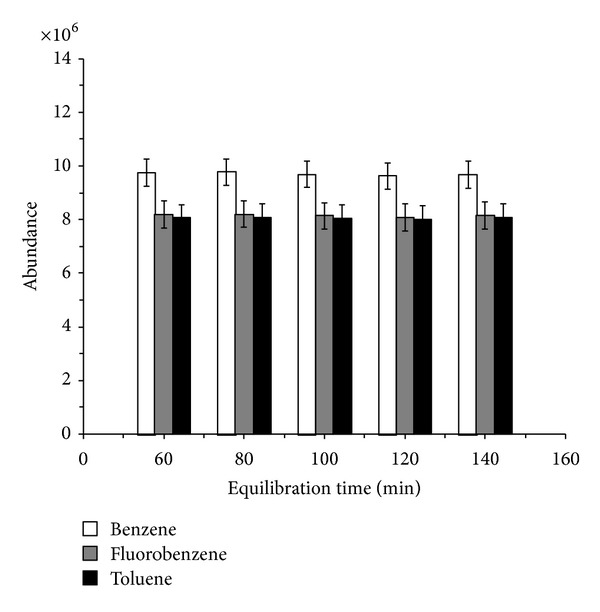
Effect of equilibration time on benzene, toluene and IS peak abundance (equilibration temperature: 120°C).

When the incubation temperature was set to 120°C, the effect of five equilibration times of 5, 10, 15, 20, and 25 min on headspace analysis was screened. Our finding demonstrates that vapor-liquid equilibrium of the analytes and IS can be achieved within 5 min at the given temperature (120°C). Equilibration times longer than 5 min do not yield the expected increase in signal (Figure 4). Long-time incubation under high temperature will pose potential decomposition risk for complex samples. For quick and effective determination of benzene and toluene in pesticide EC, 120°C and 5 min were used as optimum headspace conditions. 

### 3.3. Linearity

Accurately weighed benzene (32, 66, 85, 97, 120, 136, and 160 mg) and toluene (36, 64, 83, 105, 125, 141, and 160 mg) to 10 mL of volumetric flask, spiked with 1.0 mL of stock IS solution each were, made up to constant volume with stock solution A to afford calibration standard solutions. Under the optimal headspace and chromatographic conditions, separate calibration curves of benzene and toluene were prepared by plotting relative peak area ratios of benzene and toluene to IS against the analytes concentration. The response linearity within the 3.2–16.0 g/L range for benzene and 3.6–16.0 g/L range for toluene was good, and it was characterized by a correlation coefficient of 0.998 and 0.997, respectively. The regression equations for benzene and toluene were as follows: *y* = 0.095*x* + 0.2387 (*n* = 7, *R* ^2^ = 0.9983) and *y* = 0.1057*x* + 0.1848 (*n* = 7, *R* ^2^ = 0.9969), where *y* stands for peak area ratio of analyte to IS and *x* represents concentration of analyte in sample (in g/L). 

### 3.4. Recovery and Precision

The recovery and precision tests of this analytical procedure were performed by spiking blank different matrix solutions with accurate benzene and toluene. The general procedure was as follows: accurately weighed benzene and toluene to 10 mL of volumetric flask, spiked with 1.0 mL of stock IS solution each, were made up to constant volume with different stock solutions to afford spiked solutions for tests. Five replicates were analysed for each spiking level. To our delight, as shown in Table 1, the recovery and precision (RSD%, *n* = 5) of the method for toxic benzene determination were very satisfactory. For another analyte toluene, the results were generally satisfied.

### 3.5. Matrix Effect

The matrix effect is a very common case encountered in HSGC analysis, since the matrix markedly influences the vapor-liquid equilibrium of the analyte in the headspace of closed vial. The diversity of the active ingredient, organic solvent, and surfactants makes considerable complex matrix for pesticide EC. In our previous report for HSGC determination of methanol in pesticide EC, the recovery was not acceptable for different matrix under low equilibration temperature [14]. In the present paper, for different matrices including pure solvent (xylene), 40.0% (w/w) chlorpyrifos EC (stock solution A), 40.0% (w/w) chlorpyrifos EC without surfactant (stock solution B), 10.0% (w/w) Tween-80 solution (stock solution C), and 2.5% (w/w) beta-cypermethrin EC (stock solution D), the good recoveries of benzene and toluene clearly demonstrate that the matrix effect is basically eliminated under high equilibration temperature (120°C), and the developed methods are suitable for a variety of pesticide ECs. 

## 4. Conclusions

Integrating the advantages of both automated headspace and fast GC-MS, a simple, reliable, and efficient HSGC-MS method was established for rapid simultaneous determination of benzene and toluene in pesticide EC. By choosing a very small sample size (50 *μ*L) and a high incubation temperature (120°C), a near-complete mass transfer of benzene and toluene from the liquid sample to the vapor phase was achieved within 5 min. More importantly, the matrix effect can be basically eliminated under the optimized conditions, which renders the present method applicable for all kinds of pesticide EC, even for other liquid pesticide formulations quality control or sample screening. When the concentrations of benzene and toluene in pesticide EC fall into the range 3.2–16.0 g/L, the regression equation can be used directly. If the concentrations are beyond 16.0 g/L, dilution of the formulation with xylene can be adopted to expand the application scope of the developed method, since the xylene as a matrix has little influence on the assay of benzene and toluene as shown in Table 1. 

## Figures and Tables

**Table 1:** The results of accuracy and precision (RSD%) studies for different matrices spiked with accurate benzene and toluene.

Analyte	Matrix	Spiking level (g/L)	Recovery range (%)	Recovery mean (%)	RSD%^a^
Benzene	Xylene	10.9	99.5–103.9	102.5	1.9
	Stock solution A	9.9	101.3–104.1	102.6	1.0
	Stock solution B	10.1	100.6–103.0	101.8	1.1
	Stock solution C	10.7	102.0–103.1	102.7	0.4
	Stock solution D	10.7	102.0–102.7	102.3	0.3

Toluene	Xylene	10.2	93.6–109.2	100.1	8.1
	Stock solution A	10.5	101.2–118.4	108.7	6.7
	Stock solution B	10.3	99.1–117.1	109.5	6.7
	Stock solution C	10.6	95.0–108.0	100.2	4.9
	Stock solution D	10.3	96.8–111.2	102.7	5.5

^a^ *n* = 5.
